# Enhancing life prospects of socially vulnerable youth through sport participation: a mixed methods study

**DOI:** 10.1186/1471-2458-14-703

**Published:** 2014-07-09

**Authors:** Sabina Super, Niels Hermens, Kirsten Verkooijen, Maria Koelen

**Affiliations:** 1 Wageningen UR, Department of Social Sciences, Health and Society Group, P.O. Box 8130, 6700 EW Wageningen, the Netherlands

**Keywords:** Positive youth development, Self-regulation, Salutogenesis, Sport

## Abstract

**Background:**

Sport participation has been associated with improved life prospects such as academic performance and employability prospects. As such, promoting sport participation might be a way to increase life prospects, especially for socially vulnerable youth because they are less physically active than their peers. However, the evidence for the causal effect of sport participation on these outcomes is still limited and little is known about factors that play a role in this possible effect. The aim of this study is four-fold. First, the causal effect of sport participation on life prospects is studied and the underlying mechanisms of this relation are explored. Secondly, the life experiences of the youngsters in the sport context, that may contribute to skill development, are studied. Thirdly, social conditions for a positive effect are explored, as sport is likely to have a positive effect under specific conditions. Fourthly, this study aims to provide insights on the elements of successful partnerships between youth care organisations and local sport clubs.

**Methods and design:**

This protocol reports on a mixed method study. An intervention that aims to increase the sport participation of socially vulnerable youth, between 12–23 years old, is implemented in three regions of a Rotterdam youth care organisation. The youngsters in the two control regions receive care-as-usual. The main outcome variables, collected via questionnaires, are the life prospect, sense of coherence and self-regulation skills of the youngsters after 6 and 18 months of follow-up. The Motivational Climate Scale is administered to explore the social conditions for a positive effect and interviews are conducted with sport coaches to explore their role in skill development. Interviews with the youngsters are conducted to gain insight on the life experiences that may lead to skill development. The elements of successful partnerships are collected during interviews with youth care professionals, sport coaches and other stakeholders in the sport context.

**Discussion:**

The results of this study can support efforts of youth care organisations and local sport clubs to improve the life prospects of socially vulnerable youth through sport participation.

**Trial registration:**

Trialregister.nl NTR4621 Date of Registration: 2 June 2014

## Background

Sport participation is associated with many positive outcomes and is advocated by health professionals and policy-makers to combat problems in various societal domains (e.g. health, education and participation) [1,2]. Hence, physical activity and sport participation have been used in what Coalter calls the evangelic policy rhetoric [3], as a panacea to social problems [1], particularly to improve life prospects of socially vulnerable youth [4,5]. Scientific studies indeed show a positive relationship between sport participation and many beneficial outcomes, such as social inclusion [6], pro-social behaviour [1], academic achievement [7,8], and social and emotional well-being [2]. However, sport-based activities are often treated as a black box, ‘magically’ producing positive outcomes [9,10]. There is still uncertainty about the causal relationship between sport participation and the personal development of young people and the processes occurring in the ‘black box’ that may lead to improved life prospects. This study aims to fill some of these knowledge gaps.

First, this study aims to address the causal effect of sport participation on life prospects. Bailey distinguishes five domains of beneficial outcomes of sport participation for young children: physical, lifestyle, affective, social and cognitive outcomes [11]. The evidence for the causal effect of sport on these domains varies, for example the effect of sport on physical outcomes is well-established whereas the effect on cognitive outcomes is less researched [11]. In addition, little is known about how sport participation may lead to these positive outcomes and which factors play a role in this positive effect [1,11,12]. One interesting line of research is the idea that sport participation may contribute to skill development and that these skills can be transferred to other societal domains [13,14]. Although several researchers have suggested that skills accumulated in sport can be transferred to other domains, no study actually tested this idea [15]. Taking a longitudinal approach, this study aims to explore the positive effects of sport participation for socially vulnerable youth and the underlying mechanisms.

Second, this study aims to address the processes that take place in the sport context that could enhance the personal development of socially vulnerable youth [2]. Coalter argues that a focus on the experiences of sport participation is important for understanding how sport participation may produce positive outcomes [9,10]. “*It is probably not the mere participation in sport that enhances positive development but the individual’s experience in sport that may be the critical factor* (p. 247) [16]”. This study, therefore, examines the life experiences of youngsters in the sport context to provide a deeper understanding of the critical factors in the sport experiences of socially vulnerable youngsters for their personal development.

Thirdly, the social conditions that can optimise the effect of sport participation on life prospects are explored. Many researchers have warranted that sport does not produce positive outcomes per se [12,17,18]. A positive socio-pedagogical climate has been found a very important social condition [12]. However, these social conditions are often studied in qualitative studies and they require more empirical testing, also with specific attention to these social conditions in different groups, settings and practices [12].

Fourthly, this study investigates the coordinated action of youth care organisations and local sport clubs that is required to include sport-based activities in youth care practices [18,19]. Partnerships between these organisations are no sinecure, because youth care organisations and local sport clubs differ on several aspects such as organisational missions, working hours, and type of representatives (i.e. professionals versus volunteers). Existing research into coordinated action in the public health sector indicates that factors related to the organisation of a partnership help to deal with such organisational differences [20]. Nevertheless, few studies examined the performance of coordinated action [21], especially concerning coordinated action between professionals and volunteers of different organisations. This study aims to explore and examine what factors, related to the organisation of partnerships between professional youth care organisations and voluntary sport clubs, improve the performance of coordinated action between these organisations.

In sum, research conducted on the positive outcomes of sport for young people seems to suggest that youngsters can benefit from sport participation and that their life prospects may improve by engaging in sport and physical activity. This seems to hold especially true for socially vulnerable youth because they participate less in sport than their average peers [22]. However, to support the claim that sport participation leads to improved life prospects more research is warranted. Consequently, this study aims:

1. To provide insights into the causal relationship between sport participation and life prospects of socially vulnerable youth and the underlying mechanism by which sport can improve life prospects.

2. To explore the life experiences in the sport context of socially vulnerable youth that can lead to skill development.

3. To examine the social conditions that may strengthen the positive effect of sport on life prospects.

4. To provide insights into the organisational context of successful sport inclusion in youth care practices by exploring elements of successful and enduring partnerships between professional and voluntary organisations.

### Theoretical framework

The following paragraphs discuss the theoretical framework of the study, starting with a short description of the positive youth development paradigm as this serves as a theoretical basis for the study. In subsequent paragraphs, theory and research findings related to the four study aims are discussed in more detail.

### Positive youth development through sport

The positive youth development paradigm takes as its starting point the potential that children and adolescents hold in terms of the resources, strengths and interests they possess, rather than focusing on their problems and deficits [23]. The problem-centred approach has dominated in research on youth development for example by focusing on the prevention of anti-social behaviour, learning problems, drug abuse or psychological problems [24]. Many studies on the effects of sport participation on socially vulnerable youth adopt a problem-centred approach. A large body of research has focused on the effect of sport on anti-social behaviour and prevention of crime [19,25]. Other studies report on sports-based interventions that aim to overcome specific deficits in children. For example, a study among Swiss school children demonstrated a reduction of social anxiety among children that participated in a team sport [4]. The positive youth development paradigm acknowledges that there are children in socially vulnerable positions, but rejects the idea that social interventions are mainly an effort to ‘overcome deficits and risks’ [23]. “*The positive youth development perspective emphasises the manifest potentialities rather than the supposed incapacities of young people – including young people from the most disadvantaged backgrounds and those with the most troubled histories* (p.15) [23]”.

### Study aim 1: causal effect of sport participation and underlying mechanisms

Research from a positive youth development perspective focuses on the internal assets (e.g. social skills and positive values) and external assets (e.g. the community, meaningful others, and school) that help young people to be well-prepared for a healthy life and social and emotional well-being during adulthood. Local sport clubs can be considered environments in which internal and external assets are built, and hence are environments for positive youth development [26]. In this light, it has been hypothesised that sport participation may lead to increased assets that subsequently can be applied in other settings such as school [13-15]. Although several studies suggest that certain skills accumulated in sport can be transferred to other domains, no study actually tested this idea [15]. In this study, we investigate the longitudinal effect of sport participation on self-regulations skills and sense of coherence (SOC), and the transfer of these skills to other societal domains.

#### Self-regulation skills

Self-regulation refers to “*the processes by which the self alters its own responses, including thoughts, emotions, and behaviours*[27]”. It is considered to have a great influence on a person’s success [27], in the broadest sense of the word and in various domains such as in academics [28,29] and in sport [15]. Jonker *et al.* identified six skills that are essential in self-regulatory processes: planning, monitoring, self-evaluation, effort, reflection and self-efficacy [30]. Self-regulatory learners score high on these dimensions and are, therefore, better able to acquire knowledge and skills in different domains [30].

From a positive youth development perspective, self-regulation skills can be considered an internal asset that can help young people to get prepared for a healthy and productive adulthood. According to Posner and Rothbart [31], self-regulation is shaped in childhood by both genes and the social environment; specific exercises in this period can improve self-regulation. The sport context may be an environment in which these exercises are practiced and as such may offer opportunities for children to acquire various self-regulatory skills, which subsequently can be used in other settings [17]. A cross-sectional study conducted by Jonker *et al.* demonstrated that pre-university students that were elite athletes scored higher on planning, reflection and effort than their pre-university non-athletic peers [32]. In this study it is hypothesised that sport participation improves the self-regulatory skills of socially vulnerable youth and that this increase in self-regulatory skills translates to improved life prospects of these youngsters (see hypotheses 1, 2 and 4 in Methods/Design).

#### Sense of coherence

SOC reflects an individual’s ability to cope with difficult or stressful situations [33]. It is a core construct of the salutogenic theory, which focuses on the origins of health and well-being rather than on disease. An individual’s SOC consists of three components [34]: the idea that the stimuli from one’s environment are structured, predictable and explicable (comprehensibility); the feeling that sufficient resources are available to deal with stressors (manageability); and the feeling that the challenges are worthy of investment and engagement (meaningfulness). People with a high SOC are more likely than people with a low SOC to use Generalized Resistance Resources within the individual (e.g., self-efficacy beliefs, attitudes, self-regulation skills) or in their environment (e.g. social support, cultural stability) to combat everyday life stressors [35,36]. In this light, the amount of assets available is not the only important factor in producing positive outcomes; the ability to use the resources for this purpose is just as valuable. Increasing the SOC of socially vulnerable youth may increase the application of self-regulatory skills, which subsequently may improve their life prospects.

García-Moya *et al.* have examined the ‘constellations of contextual factors’ that lead to specific SOC-levels [37]. They conclude that the quality of parent–child relationship is the main predictor of children’s SOC, but that other contexts contribute significantly to SOC as well. In addition, they found that children could have a relatively strong SOC when a high quality parent–child relationship was absent, pointing to the compensatory effects of other contextual domains [37]. The sport context may be such a domain in which the development of SOC can take place. Interestingly, people with a stronger SOC also have been found to engage more often in physical activity [38-40]. Yet, there is no evidence on the causal relation; are people with a stronger SOC more physically active or does physical activity enhance one’s SOC? In this study, it is hypothesised that sport participation may contribute to the development of SOC and that socially vulnerable youth are better able to use their self-regulatory skills across various life domains when they have a stronger SOC (see hypotheses 1, 3 and 5 in Methods/Design).

### Study aim 2: life experiences in the sport context

As previously argued, sport in itself is not likely to produce positive outcomes [9,16]. To understand the effect of sport participation on the life prospects of socially vulnerable youth, an examination of the processes occurring in the sport context is necessary. Researchers have suggested that positive life experiences are important for personal development and that the sport context may provide room for these positive experiences [10,41,42]. However, currently, the experiences of the youngsters are often quantitatively examined, for example using the Youth Experiences Scale (YES) [10,41]. “W*hile sport might provide the context for the development of positive experiences, the social process of participation is the key to understanding what is happening* (p. 595)” [9]. An examination of the life experiences of the youngsters in the sport context could contribute this understanding of the social processes occurring in the sport context [9].

Life experiences are also an important concept in the salutogenic model. According to Antonovsky, SOC develops in childhood and early adulthood when people have life experiences that are characterised by an overload-underload balance (i.e. influencing manageability), consistency (i.e. influencing comprehensibility), and socially relevant decision-making (i.e. influencing meaningfulness) [35]. Sport participation by socially vulnerable youth may provide room for life experiences that develop SOC when these are comprehensible, manageable and meaningful [43]. This study aims to explore the life experiences of the youngsters in light of the theory of sense of coherence as this may increase our understanding of the (social) processes taking place in the sport context that could contribute to personal development.

### Study aim 3: social conditions for a positive effect

It has been warranted by many researchers that sport participation does not produce positive outcomes per se [12,17,18]. It seems that, for sport to have a positive effect on young people, certain conditions have to be met. A positive socio-pedagogical climate is one conducive factor that has been found important for generating positive social outcomes [12]. Other factors that seem to be important are the social support children receive from their parents, the training skills of the coach, the ability of the coach to maintain a motivational and safe climate and providing ample opportunities to develop skills [12,17]. Also the possibility to reflect is considered a prerequisite for the development of both self-regulation skills and sense of coherence [15]. However, these social conditions require more empirical testing, also with specific attention for these factors in different groups, settings and practices [12].

With respect to a positive socio-pedagogical climate, researchers have distinguished between an ego- and a mastery-motivational (or task-involving) climate [28,44,45]. An ego-motivational climate emphasises competition and out-performing others, whereas a mastery-motivational climate stresses cooperative learning and self-referenced improvement [44]. A mastery-motivational climate is considered to contribute to personal development. For example, a mastery-motivational climate is associated with adaptive learning strategies [28], reduced state anxiety [45] and increased self-efficacy [45]. Nonetheless, there is limited empirical evidence for a moderating effect of motivational climate in the relation between sport participation and the development of self-regulation skills and sense of coherence. We will quantitatively assess the moderating effect of a motivational sport climate in the relation between sport participation and life prospects (see hypothesis 6 in Methods/Design).

### Study aim 4: elements of successful partnerships

Coordinated action of youth care organisations and local sport clubs may help to increase sport participation of socially vulnerable youth in a sport environment that enhances their personal development. Youth care organisations and local sport clubs, however, differ on several aspects such as organisational missions, working hours, culture and type of representatives (i.e. professionals versus volunteers) [46-48]. As a result of these differences, partnerships between these organisations are no sinecure. To explore the elements that improve the quality and the performance of partnerships between youth care organisations and local sport clubs, we take the perspective of the whole network. This perspective assumes that the organisations involved in a partnership are jointly working towards a more or less common goal. Consequently, researchers taking the perspective of the whole network study the elements of the partnership that may affect this common outcome. The perspective of the whole network complements the perspective of the individual organisations that starts from the benefits the individual organisations experience from participating in partnerships [21].

There are personal and organisational factors that may frustrate or strengthen the quality and the performance of the partnership [20,49]. Personal factors of the people involved in the partnership that influence the partnership are, for instance, different attitudes towards the collaboration, different experience with participation in partnerships and different levels of self-efficacy (i.e. the feeling that they are able to influence the coordinated actions and the performance of the partnership). In addition, these people represent organisations with different values, cultures, rituals, targets and funding possibilities (i.e. organisational factors). Nonetheless, research also indicates that there are factors related to the organisation of the partnership that might help to deal with these personal and organisational differences and, thus, may improve the network’s performance [20,21,50]. The occurrence of these elements seems to be especially relevant for partnerships between professional organisations in the public sector and voluntary-based organised sport clubs because these organisations differ on many aspects. A practical example of this is that coordinated action in the public health sector is frustrated by the different opening hours of professional health organisations and voluntary sport clubs [46].

**Figure 1:**
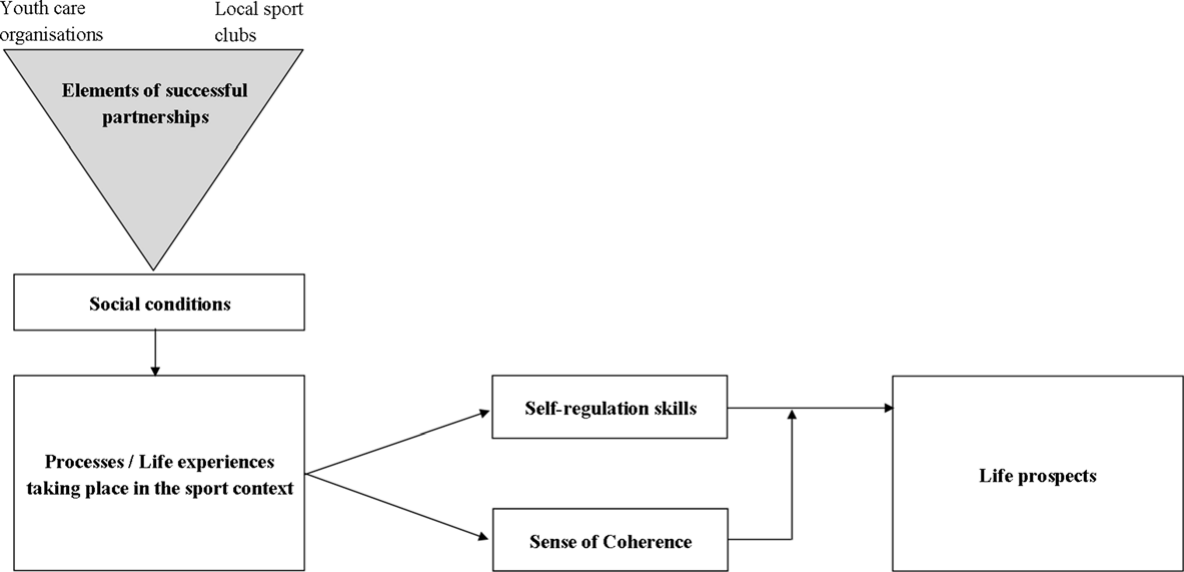
Theoretical model of the study.

The institutional and (inter)personal differences that may be brought into partnerships are described in the Healthy ALLiances (HALL) Framework [20]. Moreover, Koelen *et al.* came up with seven factors related to the organisation of partnerships that help to deal with these institutional and (inter)personal differences, and that may consequently contribute to the partnership’s performance [20]. These seven factors relating to the organisation of the partnership are a flexible time frame, clear roles and responsibilities, a clear communication structure, usage of the expertise and capacities of the involved organisations, a shared mission, visibility of (the results of) the partnership, and a neutral and empowering management of the partnership. Concerning the management of the partnership, Williams argued that so-called boundary spanners might function as a bridge between different organisations in partnerships [49]. These boundary spanners are individuals who work in collaborative environments and who possess communication, co-ordination, mediating and entrepreneurial skill to deal with tensions and differences between organisations in a partnership. Boundary spanners, for example, are initiators of collaborations, partnership coordinators and frontline workers collaborating with frontline workers of other organisations [49].A complete overview of the four study aims is presented in the theoretical model (see Figure 1).

## Methods/Design

### Study design

**Figure 2:**
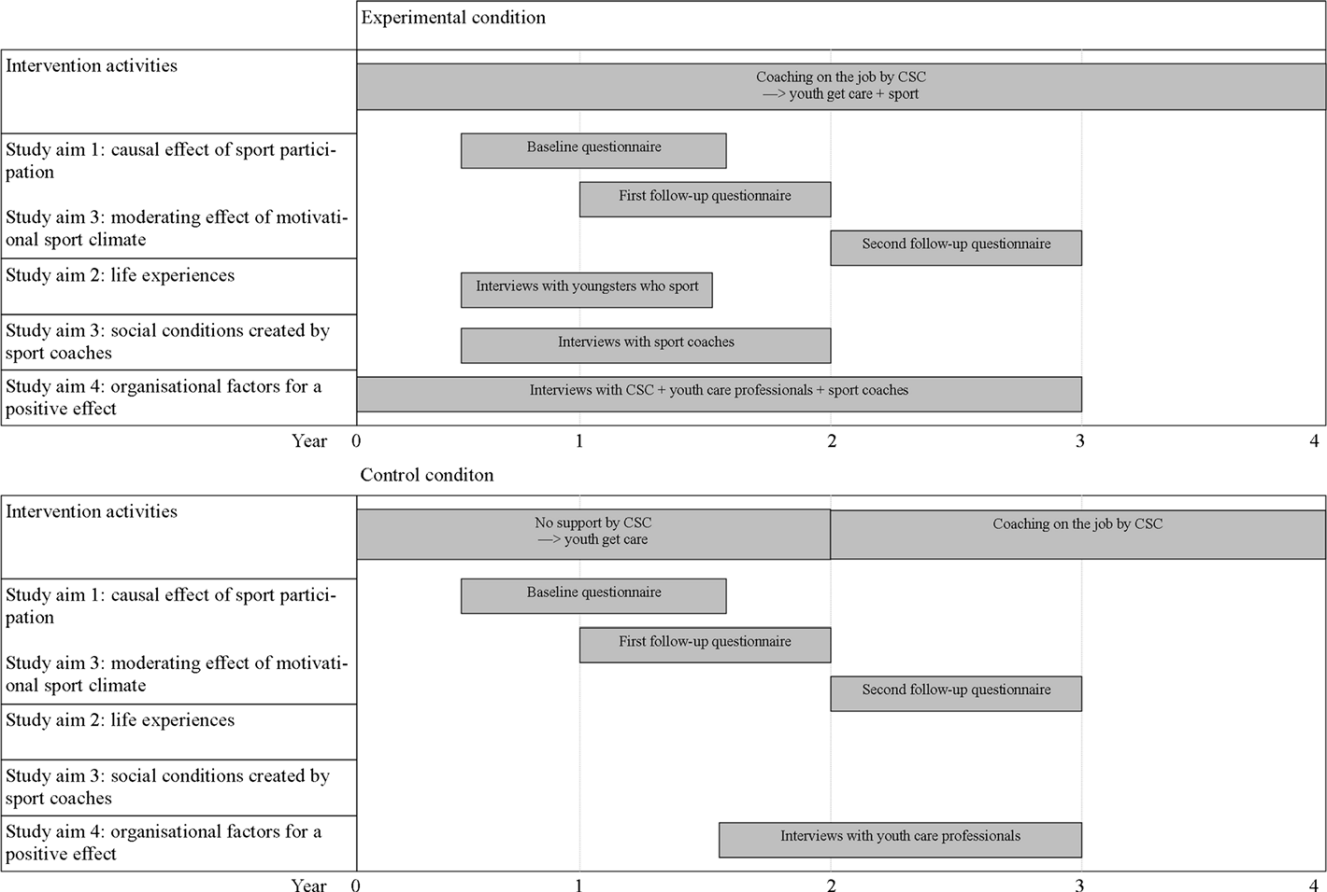
Overview data collection methods.

Quantitative and qualitative data for this study will be collected in several phases and for different purposes (see for an overview Figure 2). All the data is gathered in Rotterdam where an intervention is implemented in a youth care organisation that aims to increase the sport participation of socially vulnerable youth. The intervention will be implemented in three regions of the Rotterdam youth care organisation, two regions are in the control group where no intervention activities take place. As randomisation of the regions is not possible, the regions for the experimental and control condition are selected in such a way that the baseline characteristics of the youngsters are as similar as possible (i.e. a non-equivalent control group design). In addition, to avoid contamination, the experimental groups and the control groups are not located in adjacent regions. After describing the intervention into more detail, the data collection methods are discussed per study aim.

### Intervention

At the start of a care program at the Rotterdam youth care organisation every youth care professional sets, together with the youngster, certain goals to be reached during the care program. These goals can relate to the individual, school, work, family and positive leisure time activities. In the experimental region, specific attention is paid to sport participation as a positive leisure time activity. To increase the attention for sport as a positive leisure time activity, a Care Sport Connector (CSC) is appointed to act as a boundary spanner to connect youth care and sport. As a key-player in the network, the CSC is able to spark initiatives of collaboration between youth care professionals and organised sport clubs, and to identify local sport organisations that are capable of building conducive sport environments. The CSC will bring existing collaborations with local sport clubs to the attention of youth care professionals and will facilitate initiatives of the youth care professionals to make use of sport interventions.

It is expected that a training among the youth care professionals and collaboration with a Care Sport Connector (CSC) in the experimental group will translate to more sport participation among the youngsters of these professionals. The CSC will motivate and train the youth care professionals in the experimental condition to integrate sport activities in the care that they deliver. At the start of every care program, the youth care professional in the experimental regions are expected to inventory, together with the youngsters, the possibilities of sport participation during the care program. The youth care professionals are also expected to be involved in guiding the youngsters to local sport organisations.

Training of the youth care professionals by the CSC will exist of various activities, such as interactive workshops, individual face-to-face sessions, and provision of written (online) information. Various topics will be discussed during these events that are relevant for the youth care professionals, for example focusing on practical barriers that they may encounter when guiding their youngsters to local sport organisations (such as money constraints, ethical issues and lack of parental support). These topics were selected after conducting several interviews with youth care professionals discussing their view of the inclusion of sport in youth care practices. During the intervention, additional events may be organised that match the needs of the youth care professionals and the CSC. As such, the training of the youth care professionals can be seen as coaching-on-the-job and the activities can be adapted to individual needs. All activities that take place will be carefully monitored and recorded, so that in the end a precise description of the actual intervention can be given. The activities of the CSC will continue after the end of this study and will be expanded to other regions of the Rotterdam youth care organisation as well.

Within the experimental regions, specific attention is also paid to the socio-pedagogical climate at the local sport clubs. A pedagogue is employed in the experimental regions to support the local sports clubs in their efforts to create a positive learning environment for all children. Several elements of the socio-pedagogical climate receive specific attention such as the setting and maintenance of basic rules at the sport clubs, improving the pedagogical qualities of the trainers and increasing the involvement of parents at the sport club. Similarly to the activities conducted by the CSC, the activities of the pedagogue are documented during the intervention to be able to provide a clear description at the end of the project.

### Study aim 1: effect of sport participation on life prospects

The first study aim focuses on the causal effect of sport participation on the life prospects of socially vulnerable youth and the underlying mechanisms. More specifically, the following hypotheses were formulated:

1. Sport participation during the care program of socially vulnerable youth leads to improved life prospects as measured in: (a.) school level and performance; (b.) work attendance and performance; (c.) subjective health; (d.) well-being, and; (e.) future outlook.

2. Sport participation leads to enhanced self-regulation skills (i.e. planning, self-monitoring, effort and reflection).

3. Sport participation leads to improved SOC-levels.

4. The positive effect of sport participation on life prospects is mediated by self-regulation skills.

5. The positive effect of sport participation, via self-regulation skills, on life prospects is moderated by SOC.

#### Study design and procedure

To test these hypotheses, quantitative data will be collected at three time points during the intervention. Data will be collected at baseline (i.e. when the youngsters enter the care program), after six months follow-up (i.e. at the end of the care program and intervention) and after 18 months follow-up (see Figure 2). The data of the baseline measurement and of the first follow-up will be collected using paper and digital questionnaires administered by the youth care professional. During the second follow-up, a digital and a paper questionnaire will be send to the youngsters who have left the youth care organisation at that point. If necessary, the researchers will go door-to-door to increase the response rate.

#### Participants and recruitment

The study population are the youngsters entering the Rotterdam youth care organisation. These youngsters are between 12 and 23 years old and have some behavioural and/or developmental problems. All youngsters in the intervention and control region will be invited to join the study. Youngsters with depressive or suicidal thoughts will be excluded from this study to avoid that the questionnaires included in this study worsen the psychological well-being of the participants.

#### Informed consent

Due to the vulnerable nature of the study population, special attention will be paid to the obtainment of informed consent. This project is performed in accordance with the code of conduct for minors [51]. The information letter and the informed consent form that are given to the youngsters also have to be signed by the parents or guardian. If necessary, the youth care professional will discuss the informed consent with the parents or guardian, especially when language or educational barriers of the parents prevent them from understanding the written text. If, during the intake procedure, the youth care professional is in contact with only one of the parents, for instance in the case of a divorce, standard procedures of youth care practice will be followed. This means that the youth care professional will communicate with the parent who is registered at the youth care organisation, and will ask the parent if there is another parent who also has to give informed consent. This procedure is used by youth care organisations because they do not want to harm the possible vulnerable relationship between parents and as they have to respect the privacy of the parents. In case the second parent responds and disapproves of the child’s participation, the child will be excluded from the study. In case the second parent does not respond, the registered parent will be asked if there might be any reason to expect that the other parent disapproves of the child’s participation in the research. If this is the case, the child will be excluded from the study. This procedure has been approved by the Medical Ethical Committee of Wageningen University.

#### Measures

The following measures are included in the three questionnaires (see for an overview Table 1).

**Table 1:** Overview over measurements across the three questionnaires

**Concept**	**Indicator**	**Questionnaire T**_**0 **_**T**_**1 **_**T**_**2**_			**Instrument**
**Personal characteristics**	Sex, age, ethnic background, education, employment	X			
**Life prospects**	Subjective health	X	X	X	2 items from SF-36
	Problem behaviour	X	X	X	SDQ
	School or employment status	X	X	X	
	Functioning at school or work	X	X	X	
**Self-regulation skills**	Planning	X	X	X	Planning subscale SRL-SRS
	Self-monitoring	X	X	X	Self-monitoring subscale SRL-SRS
	Effort	X	X	X	Effort subscale SRL-SRS
	Reflection	X	X	X	Reflection subscale SRL-SRS
**Sense of coherence**	Sense of coherence	X	X	X	Orientation to Life Questionnaire (SOC-13)
**Sport participation**	Frequency and duration	X	X	X	RSO
	Type of sport	X	X	X	RSO
	Membership sport/fitness club	X	X	X	RSO
	Sport as part of treatment plan		X		
**Sport climate**	Motivational sport climate		X	X	MCSYS

*Note.* SDQ: Strengths and Difficulties Questionnaire; SRL-SRS: Self-Regulation of Learning Self-Report Scale; RSO: Richtlijn Sportdeelname Onderzoek; MCSYS: Motivational Climate Scale for Youth Sports.

#### Life prospects

The primary outcome is the life prospects of the youngsters which will be assessed using four indicators. Subjective health of the youngsters will be assessed using two questions: “In general, how good is your health?” and “Compared to a half year ago, how would you rate your health now?”. The Strengths and Difficulties Questionnaire will be administered to measure behaviour, emotions and relationships [52]. Objective employment status (employed or unemployed) or school status (school level) will be recorded. Finally, the youngsters are asked to report how their teacher or boss would likely evaluate their work, as an indicator of school or work performance.

#### Self-regulation skills

The self-regulation skills will be assessed using the Self-Regulation of Learning Self-Report Scale [30]. The original scale consists of six subscales of which four will be used in this study. The selection is based on the relevance of the different sub-scales for the purpose of this study. The following scales will be included: planning, self-monitoring, reflection and effort.

#### Sense of coherence

Sense of coherence will be estimated using the Orientation to Life Questionnaire (SOC-13) adapted specifically for youngsters [53].

#### Sport participation

Measurements regarding sport participation will be based on the Dutch Guideline for Sport Participation Research (Richtlijn Sportdeelname Onderzoek (RSO)) with recall-periods adapted to fit the time frame of this research [54]. The items included in the questionnaire address the frequency and duration of sport activities, the type of sport activities, the membership of sport/fitness club and the inclusion of sport in the treatment plan.

#### Sample size

A power analysis using G-power has been carried out to determine the minimal sample size. The required number of participants (with the number of measurements set at 3) to find small effects (f 0.20) is 244, for a significance level of 5% and a power of 0.80. Allowing for a drop out of 20% at time 2 and 30% at time 3, a total baseline sample of 436 participants is required. The present design is expected to result in a total sample of 600 participants.

#### Data analysis

Quantitative data will be analysed with the use of SPSS software (PASW statistics). The data will be analysed using multilevel modelling techniques. Single-level multiple regression models ignore the complexity of the hierarchical data within this current study and are likely to produce underestimated variances and standard errors. Multilevel modelling is especially suitable for repeated measurements of individuals nested in groups, as it is capable of examining correlated data and unequal variances [55]. In addition, because we have an unbalanced design, multilevel modelling will produce efficient estimates of the model parameters and covariance components. Within our study, the repeated measures (level 1 unit) of the individuals (level 2 unit) are nested in a region of the youth care organisation (level 3 unit). Hence, the observations are not independent from one another, because the individuals within one region are likely to be more similar as compared to other regions (i.e. between regions). For ordinal outcomes, generalized linear mixed models will be used to accommodate the different probability distributions of the data, as compared to continuous outcomes.

### Study aim 2: Life experiences of socially vulnerable youth in sport context

#### Study design and participants

Interviews will be conducted with youngsters in the experimental regions during their time at the Rotterdam youth care organisation. The youth care professionals will be asked to identify youngsters for whom sport participation seems to work out well (rich response sampling) [56]. Data is collected until data saturation is reached. In-depth interviews will be conducted that aim to understand the life experiences of the youngsters in the sport context that may contribute to enhanced life prospects. The interviews will be very open and aim to invoke narratives of the youngsters about their sporting activities. Investigating the experiences of the youngsters may enhance our understanding of how the social interaction (with sport coaches and peers) in the sport context may lead to improved life prospects. Czarniawska points out that narratives are *“…often substituting chronology for causality”* (p. 651) [57]. The data gathered via these in-depth interviews cannot provide evidence on the causal relation between sport and life prospects in a statistical sense. However, the data can provide insights on the meanings that youngsters attach to activities in the sport context and the perceived role these activities play in their personal development. The interviews will be audio-taped and transcribed intelligent verbatim style.

#### Data analysis

The transcripts of the interviews will be coded and analysed using software for qualitative data analysis (Atlas.ti). The data will be analysed following the hermeneutical approach which will allow us to review the experiences of the participants in their own words [58]. The hermeneutical approach emphasises interpretation of phenomena as articulated by the interviewees and, more importantly, interpreted by the researcher. Reaching understanding of these phenomena occurs in a cyclical process in which the researcher moves from the whole text to parts of the text, and vice versa. Parts of the text cannot be understood without looking at the whole text, and the whole text only makes sense when looking at specific parts of the text.

The experiences of the youngsters will be explored in the light of positive youth development theory and Antonovsky’s sense of coherence. Through reading the transcripts and interpreting the described phenomena, the experiences of the youngsters can be contextualised and understood in light of these theories. The hermeneutical approach consists of a dialogue between the researcher and the text and, hence, the understanding of phenomena is always biased [59,60]. The aim of the hermeneutical study is then to provide one unique and partial understanding of how youngsters experience sport activities in relation to their personal development.

### Study aim 3: social conditions

To study the role of social conditions in the effect of sport participation on life prospects, two different research approaches are adopted. The first approach quantitatively assesses the moderating role of a motivational sport climate in the relationship between sport participation and life prospects. The second approach qualitatively examines the role of the sport coaches in creating social conditions that could improve the personal development of socially vulnerable youth.

### Quantitative sub-study: the moderating role of motivational sport climate

To study the influence of a motivational sport climate on the relation between sport participation and life prospects, a scale measuring sport climate will be included in the questionnaires administered to assess the causal effect of sport participation (i.e. study aim 1). The Motivational Climate Scale for Youth Sports (MCSY) contains two subscales for a mastery-motivational and an ego-motivational climate and assesses the youth’s perceived motivational sport climate. This scale will be included in the second and third questionnaire administered at, respectively, 6 months and 18 months of follow-up [44]. The following hypothesis (hypothesis 6) was formulated: The positive effect of sport participation on life prospects is moderated by motivational sport climate. The moderating effect of motivational sport climate will be statistically analysed using multivariate regression techniques (multi-level modelling).

### Qualitative sub-study: the role of sport coaches in creating positive social conditions

#### Study design and participants

Interviews will be conducted with sport coaches about their coaching practices to see how they create situations in which positive life experiences can arise for socially vulnerable youth. A list of sport coaches will be created after consultation with the youth care professionals who are asked to keep track of the sport clubs their youngsters are joining. Sport coaches will be selected from this list in such a way that they represent a wide variety of sport types. All the interviews will be conducted within the Rotterdam context with sport coaches that give training to the youngsters of the youth care organisation. The interviews will be semi-structured and conducted with the purpose to reveal how sport coaches engage in interaction with the youngsters of the youth care organisation. The coaches will be invited to share their experiences of training these youngsters. In addition, specific questions are asked that address the three SOC-components. This will allow us to review the role of the sport coach in creating life experiences that could lead to enhancements in SOC. The interviews will be audio-taped and transcribed intelligent verbatim style.

#### Data analysis

Transcripts of the interviews will be used for qualitative analysis in Atlas.ti. A thematic analysis will be conducted on the data following the guidelines of Braun and Clarke [61]. They propose six phases for a thematic analysis starting with familiarising yourself with the data and generating initial codes. The codes are then ordered in themes and rearranged to generate a thematic map of the data. In the final phase of the ongoing analysis, the themes are reviewed in relation to the whole text and the literature. The analysis will be deductive in nature, based on the sense of coherence theory. Themes will be established *a priori*, based on the current literature about the SOC theory, and mapped on the data. In addition, codes not fitting with one of the pre-designed themes will be examined to see if they can form a new theme.

### Study aim 4: elements of successful partnerships

#### Study design and participants

To identify elements of successful partnerships between youth care organisations and local sport clubs, qualitative data will be collected throughout the study by conducting in-depth interviews with youth care professionals, the Care Sport Connector (CSC) and stakeholders in the local sport clubs. At least five interviews with randomly selected youth care professionals and five interviews with randomly selected stakeholder in the organised sport clubs will be conducted in both the intervention and the control area. Three interviews with the CSC will be employed in the intervention area. Additional interviews will be conducted if data saturation is not reached after this number of interviews. The interviews will be audio-taped and transcribed (intelligent verbatim style).

In the interviews with the youth care professionals, the stakeholders in the local sport clubs and the CSC, the HALL-framework will be used as a starting point. Consequently, the participants will be asked about personal and organisational factors that might influence the quality and performance of the partnership, and factors related to the organisation of the partnership that may help to deal with personal and organisational differences. In addition, the researchers ask participants about the goals of the partnership, to what extent they think they have reached these goals and what factors helped to reach these goals. As a result, it is possible to investigate associations between the occurrence of specific factors (personal factors, institutional factors, and factors on the level of the organisation of the partnership) and how the participants involved in the partnership perceive the results of the partnership.

#### Data analysis

The data analysis will take place in three stages [62]. At first, the researchers will read all the data and will develop a codebook in which the HALL-framework functions as a starting point. Hence, a deductive coding approach is used in which the codes in the codebook refer to the three different factors of the HALL framework: the personal factors, the institutional factors and the factors related to the organisation of the partnership. A fourth element of the codebook will be the perceived performance of the partnership. Reading all the data before coding makes it possible to create codes for specific results of the partnership, as well as to expand the codebook with personal factors, institutional factors and factors relating to the organisation of the partnership that are not yet part of the HALL-framework.

In the second stage, the data will be analysed and presented in tables and summaries. These tables and summaries will represent the frequency participants mention specific factors in the partnership between the youth care organisation and the local sports clubs, and what the participants perceive as the performance of the partnership. To investigate which personal factors, institutional factors and factors relating the organisation of the partnership determine the performance of the partnership, associations between the occurrence of the different factors and the performance of the partnership will be explored in Atlas.ti. The presented findings will serve as a starting point for the third phase: validation of the data. Validating the data will occur by four of the nine validity procedures presented by Creswell [62]. First of all, to avoid researcher bias, two researchers will be involved in the process of data coding and data analysis. Secondly, after analysing and representing the findings, all the data will be read to search information that disconfirms the evidence. Thirdly, the researchers will confront the participants with their interpretations via e-mail or via a focus group meeting. Through this so-called member checking, participants can confirm if the researchers accurately represented their reality. Fourthly, all research activities and decisions made throughout the research process will be documented. A researcher who is not closely involved in the research project will be asked to monitor and review this documentation.

## Discussion

This study will provide a deeper understanding of sport as a tool for the personal development of socially vulnerable youth. This study has four research aims: (1) to provide insights on the causal relationship between sport participation and life prospects of socially vulnerable youth, and the underlying mechanism by which sport can improve life prospects; (2) to explore the life experiences in the sport context of socially vulnerable youth that can lead to skill development; (3) to examine the social conditions that may strengthen the positive effect of sport on life prospects, and; (4) to provide insights into the organisational context of successful sport inclusion in youth care practices by exploring elements of successful and enduring partnerships between professional and voluntary organisations.

Sport participation is linked to many positive outcomes and is often applied as a tool for positive youth development. Coakley argues that these claims of positive youth development through sport participation are voiced by ‘sport evangelist’ and are based on wishful thinking and personal testimonials [63]. As a consequence, Coakley claims that more critical research and theory is needed that could support policy-makers and program officials in making decisions about sport for positive youth development [63]. In a similar way, Coalter states: *“What is required is a developmental approach based on the de-reification of ‘sport’, and a concentration on understanding the social processes and mechanisms that might lead to desired outcomes for some participants or some organisations in certain circumstances* (p. 311)”. The current study adopts such a critical approach to sport for positive youth development by reviewing the phenomenon from different theoretical perspectives, with different methodological approaches and by examining different elements of the ‘black box’.

### Strengths and limitations

There has been a large amount of research investigating the positive outcomes of sport participation. These studies often draw conclusions on the association between sport and the studied outcome based on cross-sectional data. This current study aims to contribute to these findings by investigating the outcomes over a longer period of time (1.5 year) and to report on the causal effects of sport participation by comparing the outcomes of the experimental and the control group. We will explore the life experiences of socially vulnerable youth in the sport context which will allow us to draw a more comprehensive and rich picture of sport-based activities for socially vulnerable youth. In addition, the social conditions and elements of successful partnerships are studied to guide future initiatives of youth care organisations and local sport clubs to use sport as a tool for personal development. The mixed methods design of this study provides insights on the processes occurring in the black box of sport participation that may contribute to the personal development of socially vulnerable youth.

Most studies investigating the factors that influence the performance of the partnership operationalise this performance as the perceived performance by the participants. To explore this relationship for partnerships between youth care organisations and local sport clubs (research aim 4), we use a similar approach. As this part of our study takes place in the same context as the study activities to address research question 1–3, we will also be able to add objective measurements of the partnership’s performance such as the number of youngsters guided to local sport clubs and the presence of beneficial social conditions at local sport clubs.

This study has several limitations. First of all, the quantitative part of this study has a non-equivalent control group design. The training and support given by the Care Sport Connector (CSC) is organised on a regional level, making randomisation at the individual level of the youngsters impossible. This means that the study may be subject to selection-bias if the youngsters differ on baseline characteristics between the control and the experimental groups. Secondly, due to the voluntary nature of sport participation as part of the treatment plan it is likely that the youngsters who join a sport club differ significantly on certain characteristics from the youngsters that choose not to join. This may distort the effect measurement of sport participation on life prospects, self-regulatory skills and SOC. A third limitation concerns the fact that the intervention is executed on the level of the CSC. The effect of the intervention needs to percolate to the level of the health care professionals and the youngsters, which may reduce the effect of the intervention on the outcome measures. A large sample size is needed to measure changes in life prospects of the youngsters. Fourthly, increasing the sport participation of the youngsters in the experimental groups of the Rotterdam youth care organisation is a big challenge. These youngsters are having difficulties in one or more life domains (e.g. family, school or work) and as such are not necessarily focused on engaging in sports in their leisure time. In order to be able to find significant effects of a small size, we need a large sample. Especially the administration of the third questionnaire, when the youngsters have often left the youth care organisation, will be time-consuming for the researchers. Incentives for completion of all three questionnaires are introduced to increase response rates.

### Implications for practice

The results from this study will be used to support stakeholders in the youth care and sport context in their effort to improve the personal development of socially vulnerable youth through sport participation. An intervention protocol will be developed for relevant stakeholders that seek to integrate sport activities in youth care practices. There is increasing attention in both politics and practice for the use of sport as an intervention for social problems. Findings from this study can support initiatives by providing insights on the social conditions and organisational factors of successful sport inclusion.

### Ethics approval

This study has been approved by the Medical Ethical Committee of Wageningen University (protocol number: NL47988.081.14**)** and has been registered with the Dutch Trial Register (NTR4621). Date of Registration: 2 June 2014.

## Competing interests

The authors declare that they have no competing interests.

## Authors’ contributions

NH, KV and MK have contributed to the conception of the study. All authors have been involved in the design of the study. SS has written the first draft of the study protocol. All authors have read and contributed to the revision of the manuscript. All authors read and approved the final manuscript.

## Pre-publication history

The pre-publication history for this paper can be accessed here:

http://www.biomedcentral.com/1471-2458/14/703/prepub
